# Effectiveness of a multi-model teaching strategy to train emergency medicine residents to use point-of-care ultrasound (POCUS) for assessment of shock

**DOI:** 10.1186/s12909-025-07093-y

**Published:** 2025-04-23

**Authors:** Quanle Liu, Xintong Ma, Shuang Li, Zunjiang Li, Zhaofan Mo, Yihui Lin, Huazhen Xie, Banghan Ding

**Affiliations:** 1 Emergency Department, Guangdong Province Hospital of Chinese Medicine, Guangzhou, 510120 China; 2 The Second Affiliated Hospital of Guangzhou, University of Chinese Medicine, Guangzhou, 510120 China; 3 The Second Clinical College of Guangzhou, University of Chinese Medicine, Guangzhou, 510120 China; 4 The First Clinical College of Guangzhou University of Chinese Medicine, Guangzhou, 510120 China; 5 Zhuhai Hospital of Guangdong Provincial Hospital of Traditional Chinese Medicine, Zhuhai, 519000 China

**Keywords:** Multimodal teaching method, Point-of-care ultrasound, Standardized training of residents, Emergency teaching

## Abstract

**Objective:**

To evaluate the effectiveness and feasibility of a multimodal teaching method to train emergency residents to use point-of-care ultrasound (POCUS) in the assessment of shock.

**Methods:**

The study subjects were Emergency Medicine residents at the Second Affiliated Hospital of Guangzhou University of Chinese Medicine between January 2023 and December 2023. These residents volunteered for the study and were randomly divided into either the teaching reform (TR) group or the traditional teaching (TT) group. The assessment outcomes of the study included the residents’ scores on theoretical tests and practical tests and the residents’ satisfaction with and evaluation of the teaching method.

**Results:**

A total of 100 residents participated in this study in either the TR or TT groups. Compared with the TT group, the TR group achieved higher scores on both the theoretical test and the practical assessment (*p* < 0.05). Similarly, analysis of the questionnaire indicated that the TR group was more satisfied with their training and evaluated it higher than the TT group (*p* < 0.05).

**Conclusion:**

Integrating point-of-care ultrasound with a multimodal teaching method in standardized training for emergency medicine residents could effectively improve the teaching effect and quality, which may provide important value in the emergency teaching of residents.

## Introduction

In emergency medicine, effectively managing shock, a critical condition marked by hemodynamic instability and impaired organ perfusion, poses significant diagnostic and therapeutic challenges, and rapid intervention is often needed to prevent multiorgan dysfunction or failure [1]. Shock presentations make up 0.4–1.3% of emergency department (ED) cases and constitute nearly one-third of intensive care unit (ICU) admissions [2]. Given its varied forms—hypovolemic, cardiogenic, distributive, and obstructive—each type of shock requires distinct treatment and evaluation approaches due to differing underlying mechanisms [3]. Point-of-care ultrasound (POCUS) has become invaluable in ED teaching and training for shock assessment [4], offering rapid, repeatable, and accurate insights into cardiac function and fluid status [5], which are essential for guiding timely clinical decisions [2, 6].

Despite the extensive knowledge of the physiology and pathophysiology of shock, translating theoretical knowledge into clinical practice remains challenging for many healthcare professionals, particularly for residents who are in the phase of applying medical theory to practice. Emergency residents often report difficulties in integrating the complex physiology, anatomy, and pathophysiology of shock with real-world clinical decision-making [7]. The rapidly evolving nature of shock requires a precise approach to diagnosis and treatment, and the gap between textbooks and clinical practice is especially apparent, making it a challenge for residents in the emergency department under time-sensitive decision-making pressures in critical care environments [8]. Moreover, a study revealed that single-day emergency ultrasound training can enhance medical students’ understanding of shock and their ultrasound operation skills, but transferring these skills into clinical settings has proven to be challenging [9]. To address these problems and challenges, this study proposes a novel educational strategy to enhance clinical training for emergency residents. The multimodal teaching approach, termed “Multimodal Visualization- Mind Mapping-Blended-POCUS”, combines visual learning techniques with practical ultrasound training to bridge the gap between theoretical knowledge and clinical practice. Multimodal visualization involves the use of various educational tools, such as videos, charts, and simulations, to help emergency residents better understand the complex anatomy and physiology of shock [10]. Mind mapping encourages residents to organize their knowledge in a structured and logical manner, helping them retain information and apply it more effectively in clinical scenarios [11]. Point-of-care ultrasound training integrates these cognitive tools with practical bedside ultrasound skills so that residents can not only understand the theoretical aspects of shock but also visualize its manifestations in real time. This comprehensive approach aims to improve residents’ diagnostic abilities and decision-making skills, allowing them to manage shock and other critical conditions more proficiently.

This study aims to evaluate the effectiveness of this multimodal teaching method in the standardized training of emergency residents. By assessing the outcome and satisfaction of the multimodal teaching method, this study seeks to provide new insights into emergency medicine teaching strategies, helping cultivate highly skilled clinicians who are proficient in the latest techniques of using POCUS to provide precise treatment for critically ill patients.

## Methods

### Study setting

This study was a randomized controlled trial with two treatment groups: a Teaching Reform group (TR) and a Traditional Teaching group (TT). The entire trial was conducted at the Emergency Department of the Second Affiliated Hospital of Guangzhou University of Chinese Medicine from January 2023 to December 2023. The teachers involved in the project were senior emergency physicians, each holding an ultrasound certification issued by recognized academic organizations in the field of critical care, with over five years of professional experience. All patients provided written informed consent before they agreed to participate. This study was approved by the Ethics Committee of Guangzhou University of Chinese Medicine, and the number is ZM2024–385.

### Study participants

All of the residents assigned to the Emergency Department of the Second Affiliated Hospital of Guangzhou University of Chinese Medicine between January 2023 and December 2023 voluntarily served as study participants. They were randomly selected into two groups: a teaching reform group (TR) and a traditional teaching group (TT). All participants had completed foundational courses in basic and clinical medicine prior to the study.

### Teaching methods

#### TT group: Traditional lectures and bedside teaching plus case discussion

**Fig. 1:**
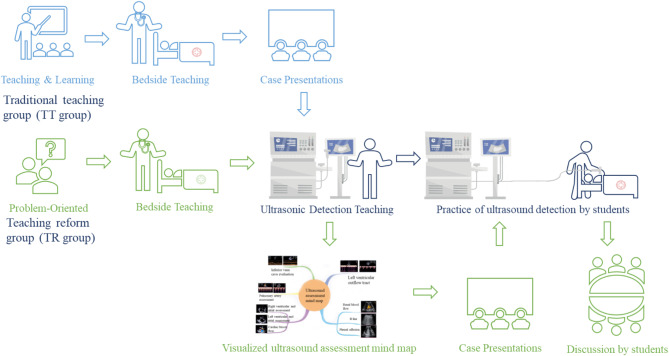
Teaching methods used in the two groups

① Before class, teachers prepared slides about shock according to the syllabus, and residents previewed the textbooks in advance. Then, the teachers delivered knowledge in a traditional classroom setting to the residents, provided bedside instruction and discussed the cases with the residents via heuristic teaching methods. After class, teachers distributed the current guidelines for assessment and management of shock to the residents for review, including “Chinese emergency septic shock clinical practice guidelines (2016)”, “Surviving Sepsis Campaign: International Guidelines for Management of Sepsis and Septic Shock 2021” and so on [12–14].,

② Combined with video teaching and the guidance of the teacher, residents used the phased probe to scan the ultrasound equipment simulator and practice on the ultrasound simulator. Each resident practiced twice with the teacher’s guidance (Fig. 1).

③ During the clinical teaching process, teachers adopted traditional demonstration methods to perform scans for patients who needed emergency ultrasound. The resident physicians subsequently observed and performed scans under the guidance of the teaching teacher.

#### TR group: Multimodal visualization-mind mapping-blended-POCUS

① Before class, the lecturers distributed guidelines and slides on the syllabus to the residents for advanced review, and the residents previewed in a blended teaching method, including acquiring knowledge of shock and ultrasonic hemodynamics online and developing a mind map based on the knowledge what they had learned offline. The instructors guided the residents in using a combination of graphics and text to create mind maps, organizing complex information into a hierarchical and structured system that could be modified and refined as needed. Following this, the residents engaged in group discussions to identify and address gaps in their understanding, further refining their mind maps through collaborative feedback [15].

② During class, to strengthen the interaction with residents, the teacher employed the flipped classroom format, problem-oriented method, case teaching method and visual POCUS teaching method to guide residents in discussing the common causes, pathophysiology and diagnosis of shock, and ultrasonic characteristics of different types of shock. Adopt the flipped classroom format, where residents first deliver the lecture, followed by the teacher providing evaluation, feedback, and additional corrections. In heuristic teaching methods, teachers posed thought-provoking questions related to the teaching content to create problem scenarios and stimulate residents’ curiosity. When residents encounter difficulties, teachers should provide further inspiration and guidance to encourage them to identify problems and develop the desire and motivation to solve them. Then residents observed videos that demonstrated the use of POCUS to assess patients with critical hemodynamic disorders and shock. The teacher guided the residents to refine the observation points one by one and establish clinical thinking for diagnosis and intervention decisions.

③After class, the residents would be required to sort out what they learned by complementing the mind mapping they had done and the online course. The teacher then employed a classic shock scenario to conduct bedside experiential learning with residents, guiding them through physical examinations and employing POCUS to scan fundamental anatomical planes. This approach aimed to foster residents’visual reasoning skills and deepen their comprehension of pathophysiology (Fig. 1).

The key differences between two groups’ teaching methodologies could been seen in Table 1.

**Table 1:** Key differences between two groups’ teaching methodologies

Phases	Content
**Before Class**	
**TT Group**	Residents previewed the text books and guidelines.
**TR Group**	Residents previewed the text books, slides and guidelines. After that, they should make a mind mapping.
**During Class**	
**TT Group**	Teachers delivered knowledge in a traditional way and demonstrated emergency ultrasound procedures as usual. Then they discussed the cases via heuristic teaching methods. Residents practiced on the ultrasound simulator twice.
**TR Group**	Teachers delivered knowledge with the flipped classroom format, problem-oriented method, case teaching method and visual POCUS teaching method. Residents observed videos about POCUS. Then teachers guided the residents to refine the observation points one by one.
**After Class**	
**TT Group**	None
**TR Group**	Residents complemented the mind mapping and teachers employed a classic shock scenario to conduct bedside experiential learning with residents.

### Assessment indicators

After the completion of the teaching session, we evaluated the theoretical knowledge and practical skills of the residents-in-training through assessments. The total score of the theoretical text was 100 points, including 60 points for objective questions and 40 points for subjective questions.

(1) Objective questions: All the questions were multiple-choice and focused mainly on theoretical knowledge of shock and ultrasound signs of shock.

(2) Subjective questions: The total score was 40 points, including descriptions of ultrasound images of shock (10 points), possible diagnoses on the basis of clinical information and ultrasound images (10 points), differential diagnoses on images (10 points) and examinations that need to be improved (10 points). The total score of the clinical assessment was 100 points, including evaluation (20 points), diagnosis (30 points), operational scoring (40 points), and overall evaluation (10 points).

(3) Satisfaction and Evaluation Questionnaire: The residents in the two groups evaluated the effects of teaching methods, including evaluations of teaching satisfaction and overall teaching evaluation, by anonymously completing questionnaires. The specific ratings for teaching satisfaction are as follows: 1 point-very poor, 2 points-relatively poor, 3 points-average, 4 points- relatively good, and 5 points -very good. The specific ratings for overall evaluation of the training were as follows: 1 point -unsatisfactory, 2 points -average, and 3 points -satisfactory.

### Statistical analysis

SPSS 25.0 statistical software was used for statistical analysis. The database was constructed via Epidata software, with all entries double-checked, logically validated and cleaned. Statistical analyses were carried out via SPSS version 23.0. Categorical data are presented as frequencies and percentages, with group comparisons performed via the chi-square test or Fisher’s exact test, as appropriate. For continuous variables, the results are displayed as the means ± standard deviations or medians, and group comparisons were performed via either t tests or nonparametric tests on the basis of the data distribution. *p* < 0.05 was considered statistically significant.

## Results

### Participant characteristics

A total of 100 emergency residents who participated in the POCUS training for Shock Assessment between January 2023 and December 2023 were included. There were 50 patients in the TT group (24 (48%) males and 26 (52%) females) and 50 patients in the TR group (28 (56%) males and 22 (44%) females) (*p* > 0.05). In addition to similar average ages (23.77 years vs. 22.65 years) between the two groups (*p* > 0.05), there were also no differences between the groups in terms of education level, major, or working duration (*p* > 0.05). Table 2 shows the differences in participant characteristics between the groups.

**Table 2:** Participant characteristics between groups (mean ± SD, *n*/%)

Index​	TT group(*n* = 50)	TR group (*n* = 50)	t/X^2^	*p*
Age (years)	23.77 ± 1.74	23.65 ± 1.83	0.34	0.74
Gender (%)			0.64	0.42
Male	24(48%)	28(56%)		
Female	26 (52%)	22(44%)		
Education level			0.42	0.81
Bachelor degree	18(36%)	15(30%)		
Master degree	25(50%)	27(54%)		
Doctor degree	7(14%)	8(16%)		
Major			0.09	0.77
Chinese medicine	44(88%)	43(86%)		
Integrated Chinese and Western Medicine	6(12%)	7(14%)		
Working duration(month)	10.86 ± 2.73	11.52 ± 2.81	-1.19	0.24

Note: Categorical data are presented as frequencies and percentages, with group comparisons performed via the chi-square test. For continuous variables, the results are displayed as the means ± standard deviations, and group comparisons were performed via either t tests. *p* < 0.05 was considered statistically significant

### Teaching efficacy between the two groups

As shown in Table 3, compared with the TT group, the emergency residents in the TR group achieved higher scores for interest, clinical analysis and problem-solving abilities, self-learning ability, broader knowledge, ability to apply theory to practice, and lower scores for learning burden (*p* < 0.001). Additionally, integrating POCUS with multimodal teaching was associated with a more active atmosphere, increased enthusiasm for asking questions, more opportunities for teacher‒student interaction and better overall satisfaction with the course, as indicated by higher scores in terms of these indicators in the TR group (*p* < 0.001).

**Table 3:** Teaching efficacy between the two groups

Index​	TT group(*n* = 50)	TR group (*n* = 50)	t	*p* value
1. After the point-of-care ultrasound teaching, how interested are you in bedside ultrasound?	2.78 ± 0.42	4.80 ± 0.40	-24.56	< 0.001
2. After the point-of-care ultrasound teaching, have your clinical analysis and problem-solving abilities improved?	3.42 ± 0.93	4.36 ± 0.69	-5.74	< 0.001
3. After the point-of-care ultrasound teaching, has your self-learning ability improved?	2.30 ± 0.91	4.72 ± 0.45	-16.84	< 0.001
4. Has this point-of-care ultrasound teaching helped you broaden your knowledge?	3.60 ± 0.86	4.94 ± 0.24	-10.65	< 0.001
5. After the point-of-care ultrasound teaching, do you think your learning burden has increased?	4.26 ± 0.83	2.88 ± 0.96	-7.69	< 0.001
6. After the point-of-care ultrasound teaching, do you think the atmosphere is more active than traditional classroom teaching?	2.96 ± 1.2	4.70 ± 0.61	-9.16	< 0.001
7. After the point-of-care ultrasound teaching, has your ability to apply theory to practice improved?	2.28 ± 0.73	4.96 ± 0.20	-25.07	< 0.001
8. After the point-of-care ultrasound teaching, has the enthusiasm for asking questions increased?	2.70 ± 1.02	5.00 ± 1.12	-16.02	< 0.001
9. After the point-of-care ultrasound teaching, are the opportunities for teacher‒student interaction increased?	3.18 ± 0.96	4.72 ± 0.54	-9.89	< 0.001
10. After the point-of-care ultrasound teaching, what is your overall satisfaction with the emergency and critical care technology course?	3.38 ± 0.86	5.00 ± 0.00	-13.41	< 0.001
Overall average score	2.95 ± 0.43	4.75 ± 0.16	-27.80	< 0.001

### Evaluation of the introduction of POCUS teaching between groups

Table 4 shows that the introduction of POCUS increased interest, clinical analysis and problem-solving abilities, language expression ability and the effectiveness of extracurricular assignments in the TR group (*p* < 0.001). POCUS teaching also enriched the selection of teaching content, the connection of the class and the level of textbook selection (*p* < 0.001). In terms of teaching methods, POCUS helped master key points and difficulties; improve the PPT design level, classroom teaching discipline, teaching ideas and logic and content proficiency; enrich modern teaching methods; and optimize the construction of course websites (*p* < 0.001).

**Table 4:** Evaluation of POCUS teaching between groups

Index​	TT group(*n* = 50)	TR group (*n* = 50)	t	*p* value
1. Interest in the course	1.48 ± 0.54	2.9 ± 0.30	-16.13	< 0.001
2. Have your clinical analysis and problem-solving abilities improved after the point-of-care ultrasound teaching?	1.88 ± 0.66	2.82 ± 0.48	-8.14	< 0.001
3. Selection of teaching content	1.96 ± 0.73	2.38 ± 0.69	-2.94	0.004
4. The connect of the class	1.64 ± 0.66	2.84 ± 0.37	-11.17	< 0.001
5. Teaching ideas and logic	1.6 ± 0.64	2.82 ± 0.38	-11.54	< 0.001
6. Master the key points and difficulties	1.92 ± 0.72	2.86 ± 0.35	-8.26	< 0.001
7. The interaction between teachers and students	2.08 ± 0.69	2.9 ± 0.30	-7.64	< 0.001
8. Language expression ability	2.7 ± 0.51	2.94 ± 0.23	-3.03	0.003
9. PPT design level	2.8 ± 0.40	2.44 ± 0.61	3.47	0.001
10. Content Proficiency	2.9 ± 0.30	2.76 ± 0.51	1.65	0.102
11. Level of textbook selection	2.6 ± 0.49	2.88 ± 0.38	-3.15	0.002
12. Classroom teaching discipline	2.88 ± 0.33	2.8 ± 0.45	1.01	0.314
13. Modern teaching methods	1.38 ± 0.49	3.00 ± 0.00	-23.36	< 0.001
14. Teaching operation drill status	1.64 ± 0.59	3.00 ± 0.00	-16.08	< 0.001
15. Effectiveness of extracurricular assignments	2.12 ± 0.72	2.9 ± 0.30	-7.07	< 0.001
16. Teaching features	1.46 ± 0.58	2.98 ± 0.14	-18.03	< 0.001
17. Teaching methods	1.60 ± 0.53	3.00 ± 0.00	-18.52	< 0.001
18. The construction of the course website	1.68 ± 0.51	2.4 ± 0.63	-6.21	< 0.001
Total average	2.02 ± 0.21	2.81 ± 0.11	-23.40	< 0.001

### Efficacy assessment between groups

With respect to the efficacy assessment, the results revealed that both the scores of the theoretical examinations and the operational assessment in the TR groups were significantly higher than those in the TT group (*p* < 0.01), demonstrating the effectiveness of the teaching reform. (Table 5)

**Table 5:** Efficacy assessment between groups

Index​	TT group(*n* = 50)	TR group (*n* = 50)	t	*p* value
Theoretical test score	60.58 ± 11.73	80.62 ± 7.9 5	-9.99	< 0.001
Operational assessment	68.24 ± 9.5 5	82.26 ± 7.4 4 9	-8.19	< 0.001

## Discussion

### Practice of “multimodal Visualization-Mind Mapping-Blended - POCUS”

#### Visualized teaching

The findings demonstrate that integrating POCUS with multimodal teaching methods significantly enhances the effectiveness of clinical training and improves trainees’ comfort levels. Visualization-based instruction plays an indispensable role in this process. The application of POCUS visual teaching can more vividly and intuitively display the pathophysiology of critical illness through multimedia forms such as images, videos, and animations, making the learning process concrete and interactive. Teachers can use visual ultrasound to transform abstract and complex physiology, pathophysiology, and anatomy into intuitive and simple images and use interactive visual learning methods to help residents better understand and remember the key of knowledge [16]. Two studies showed that ^,^ with the help of three-dimensional visualization methods such as 3D animation, video teaching, virtual laboratories, VR and other methods to display anatomical structure and function in orthopedic clinical teaching, have shown that students can learn anytime and anywhere, improving the convenience and flexibility of learning [17, 18]. In addition to its application in the classroom, visualized teaching plays an important role in the fields of online education and distance learning. With visual images, the POCUS teaching method could help establish “visual thinking strategies” through visual assessment [19]. Combined with multimodal teaching methods, video conferencing can stimulate students’ enthusiasm for learning, improve learning effects, and make the hemodynamics of shock more concrete [20]. One study reported that the application of visual teaching methods in orthopedic clinical teaching has significant effects and is worth promoting in critical care medicine education [21].

#### Mind mapping

A study revealed that web-based mind mapping combined with standardized patient protocols can improve students’ theoretical knowledge, communication skills and self-efficacy in clinical scenarios [22]. Mind mapping and concept maps assist students in answering their questions, and “visual mapping” presented in the form of mind and concept maps helps promote their thinking [23, 24]. The mind mapping of this research project presents shock pathophysiology and anatomy, such as the inferior vena cava, right heart, left heart, and ventricular outflow tract, in the form of mind and concept maps, which help recall the learned information for residents and acquire information quickly and accurately. Combined with the problem-oriented method, it improves reading ability and the ability to construct key words to descriptive questions in a structured and concrete way [25, 26].

#### Blended learning

Blended teaching is a teaching model that integrates traditional face-to-face teaching and online teaching. Several studies have shown that blended teaching provides students with a more flexible and personalized learning experience through traditional classroom teaching, flipped classrooms and web-based learning resources [27–29]. In this study, residents participated in discussions about POCUS, watched course videos, and completed test assignments online at any time and from anywhere, which not only improved students’ learning efficiency but also promoted interaction and cooperation among the residents. This study integrated the flipped classroom format into the teaching methodology, employing typical shock cases and adopting a problem-oriented approach to foster critical clinical thinking skills among students [30, 31]. It was structured into phases aligned with the shock treatment process of rescue, optimization, stabilization, and evacuation. By blending experiential learning and mobile learning directly at the bedside, residents were able to engage more deeply in the material, fostering a greater understanding and application of knowledge. Consequently, the adoption of a blended teaching model directs learners to conduct their learning independently, in a suitable manner and with an attitude toward in-depth learning.

#### POCUS teaching

As an extension of clinical examination and a tool for diagnosis, POCUS has become a signature bedside technology for clinicians [32]. POCUS plays a role in almost all medical specialties and is used in disciplines around the world, especially in critical care specialties [33]. POCUS effectively fills the gap between physical examination and imaging equipment, such as CT or MRI, with the characteristics of being used at the patient’s bedside to answer specific clinical questions, evaluate treatment and guide clinical decisions. Among them, POCUS has an irreplaceable position in focused cardiac ultrasound, lung ultrasound, E-fast, and volume assessment [34, 35]. Early POCUS training can enhance students’ understanding of organ anatomy and physiology, facilitate qualitative assessments of acute and critical illnesses, and consequently improve their ability to make clinical decisions. By improving students’ ability to acquire precise ultrasound images, diagnostic errors in the future can be reduced among medical students, thereby enhancing their clinical reasoning. Providing comprehensive ultrasound education to residents is a fundamental prerequisite for ensuring the delivery of high-quality clinical practice, and it should be vigorously encouraged and supported [36, 37]. This study integrated a multimodal teaching curriculum with bedside ultrasound imaging technology in an effort to enhance residents’ clinical thinking and participation in clinical practice. The results indicate that the TR group demonstrated higher levels of learning engagement compared to the TT group, suggesting that POCUS-guided teaching enhances teacher-student interaction and increases residents’ motivation. These findings underscore the significance of POCUS in improving the quality of medical education.

Several studies have shown that an increasing number of medical schools have incorporated POCUS into clinical courses and that residents have generated more demand for course training resources [38]. First, standardized training can help POCUS trainees acquire the necessary knowledge, skills, and behaviors at the beginning of their training, enabling them to reach a certain level of foundational knowledge. As new technologies have advanced deeply in the field of ultrasound, artificial intelligence, remote education, and immersive virtual reality have gradually been applied in the POCUS domain [39, 40]. The utilization of new technologies such as online learning and peer-assisted learning can enhance practical competencies, allowing the transition of bedside ultrasound from supervised to unsupervised practice [33, 41]. Importantly, the practical ability in POCUS is not necessarily proportional to the level of professional knowledge mastery. Regardless of the stage of practice, there are times when it is necessary to seek advice and guidance from a more experienced practitioner. Meanwhile, they should be interpreted with the patient’s pathophysiology dynamically and repeatedly [33].

### Evaluation of the effectiveness of multimodal teaching

The integration of constructivism, cognitive load theory, and self-regulated learning provides a powerful framework for the design and implementation of our programs, while advocating the use of multimodal learning analytics to capture and analyze disparate data sources such as performance metrics, engagement, and behavior patterns [42]. In our study, we took a similar approach to evaluate the effectiveness of our POCUS training program. Our findings are consistent with the theoretical insights provided by Giannakos and Cukurova, suggesting that a multi-model teaching strategy based on learning theory can significantly improve skill acquisition and adaptability in POCUS training. Multimodal teaching combines traditional face-to-face teaching with modern technology teaching methods. By integrating different teaching resources and methods, multimodal teaching aims to improve residents’ learning outcomes and education quality. Malhotra demonstrated the effectiveness of a multimodal approach grounded in constructivism and cognitive flexibility theory for interprofessional education [43]. Their findings support the use of diverse teaching methods, such as simulations and case-based learning, to enhance the acquisition and application of POCUS skills in emergency medicine residents. When evaluating the effectiveness of multimodal teaching, the following aspects can be considered. The first is the residents’ academic performance. We can compare the residents’ test scores, classroom performance and other data under the teaching model to judge whether there is a significant improvement. The second is residents’ learning interest and participation. We can observe in detail whether residents are more actively involved in learning activities under multimodal teaching and whether they are more interested in the course content. The third is the improvement of residents’ comprehensive abilities. Multimodal teaching emphasizes the cultivation of residents’ comprehensive qualities, such as innovative thinking and teamwork ability, which can be evaluated through daily observations and questionnaire surveys.

In this study, the theoretical and practical assessment scores of the residents in the TR group were significantly better than those in the TT group. In terms of teaching satisfaction, the results revealed that the satisfaction evaluation indicators of the TR group were better than those of the TT group. The evaluation of overall teaching revealed that the overall teaching satisfaction of the residents in the TR group was significantly better than that of those in the TT group. This shows that the application of “POCUS combined with a multimodal teaching method” in emergency training can improve the clinical skills of residents, meaning that the teaching reform method is feasible in education reform. In summary, we believe that the effectiveness of multimodal teaching in emergency residency training can be attributed to the following key factors: First, multisensory integration plays a critical role in multimodal teaching by combining visual, auditory, and tactile inputs to activate different regions of the brain, thereby facilitating deeper information processing and long-term memory retention [44]. Second, The integration of theory and practice is essential, as simulated scenarios, case analyses, and hands-on practice enable trainees to directly apply theoretical knowledge to practical situations, thereby reinforcing learning outcome [45]. Additionally, Personalized learning is a significant advantage of multimodal teaching, as it adapts to individual learning progress and characteristics by offering tailored content and pacing. This approach ensures that learners receive instruction aligned with their unique strengths, weaknesses, and preferences, thereby addressing diverse learning needs. By accommodating different learning styles and paces, multimodal teaching not only enhances engagement but also optimizes knowledge retention and skill acquisition, ultimately improving overall learning efficiency [46]. Last but not least, enhanced engagement is achieved through diverse teaching methods. For example, interactive instruction and gamified learning, stimulate residents’ interest and increase their participation and focus.

Due to its flexibility and versatility, multimodal teaching method can be applied to a wide range of scenarios, particularly in fields such as education, healthcare, vocational training, and technology development [47]. By leveraging tools such as virtual laboratories, interactive simulations, and visualization technologies, it helps learners master complex skills, understand abstract concepts, and provides ongoing professional development support. Multimodal teaching approach is particularly well-suited for undergraduate medical education, which provides personalized learning resources and adaptive teaching strategies, facilitates the sharing and exchange of educational resources, and maximizes resource utilization [48].

Combined with POCUS, the multimodal integrated teaching system in this project remains an exploratory subject. Owing to the lack of a well-designed POCUS curriculum, the popularization of POCUS training systems has not yet been fully incorporated into medical school courses. Therefore, resident physicians lack intuitive and visual tools to address clinical challenges, particularly in resource-limited areas, where they lack awareness of the importance of POCUS in the medical field. The results of this study indicate that POCUS serves as a practical tool and skill that can enrich medical residents’ learning experiences and that additional practical skills courses enhance medical residents’ capabilities, confidence, and attitudes.

### Limitations

This study evaluated the effectiveness of standardized training for residents under multimodal teaching methods, reflecting the real-world scenario of using POCUS to respond to critical and emergency situations. However, this study has certain limitations. First, residentsrecruited from various semesters of clinical training within the same medical institution presented heterogeneous knowledge reserves, potentially contributing to the inhomogeneity of teaching. Second, the implementation of short-term training courses as interventions might not adequately foster enduring learning abilities, as knowledge reserves and skill retention over time could affect subsequent clinical practice. Therefore, a strategy worth exploring is the combination of longitudinal training with periodic reinforcement. Third, the study population was relatively small, as all standardized training students must actively apply for the course, necessitating further expansion of the teaching population. Fourth, there was a deficiency in the supervision of teaching quality between the TR and TT groups, leading to the variability in the delivery and consistency of instructional methods and failing to promptly identify the problems and deficiencies in the process and put forward suggestions and measures for improvement, which necessitates continuous optimization. Finally, the scoring indicators we used to evaluate the effectiveness of course training have not been validated previously and need to be further optimized.

## Conclusion

The implementation of a multimodal teaching approach, integrating point-of-care ultrasound (POCUS) with visualized instruction, mind mapping, and blended learning, led to significant improvements in both theoretical and practical assessment scores among residents. This innovative educational strategy not only enhanced clinical skills and knowledge retention but also fosters greater resident engagement with instructors and satisfaction with the training experience, suggesting its potential as an effective model for advancing medical education in emergency care settings.

## Data Availability

The datasets used and/or analyzed during the current study are available from the corresponding author on reasonable request.

## Abbreviations

POCUS: Point-of-care ultrasound
TR group: Teaching reform group
TT group: Traditional teaching group
ED: Emergency department
ICU: Intensive care unit
